# Regulation of Local Sleep by the Thalamic Reticular Nucleus

**DOI:** 10.3389/fnins.2019.00576

**Published:** 2019-06-05

**Authors:** Gil Vantomme, Alejandro Osorio-Forero, Anita Lüthi, Laura M. J. Fernandez

**Affiliations:** Department of Fundamental Neurosciences, University of Lausanne, Lausanne, Switzerland

**Keywords:** sleep spindles, slow wave activity, delta waves, neural circuit, thalamocortical

## Abstract

In spite of the uniform appearance of sleep as a behavior, the sleeping brain does not produce electrical activities in unison. Different types of brain rhythms arise during sleep and vary between layers, areas, or from one functional system to another. Local heterogeneity of such activities, here referred to as local sleep, overturns fundamental tenets of sleep as a globally regulated state. However, little is still known about the neuronal circuits involved and how they can generate their own specifically-tuned sleep patterns. NREM sleep patterns emerge in the brain from interplay of activity between thalamic and cortical networks. Within this fundamental circuitry, it now turns out that the thalamic reticular nucleus (TRN) acts as a key player in local sleep control. This is based on a marked heterogeneity of the TRN in terms of its cellular and synaptic architecture, which leads to a regional diversity of NREM sleep hallmarks, such as sleep spindles, delta waves and slow oscillations. This provides first evidence for a subcortical circuit as a determinant of cortical local sleep features. Here, we review novel cellular and functional insights supporting TRN heterogeneity and how these elements come together to account for local NREM sleep. We also discuss open questions arising from these studies, focusing on mechanisms of sleep regulation and the role of local sleep in brain plasticity and cognitive functions.

## Introduction

Sleep serves distinct needs for the mammalian brain and the body, ranging from restorative and metabolic functions to offline memory consolidation (Rasch and Born, 2013). Sleep-like states occur also in jellyfish (Nath et al., 2017), the earliest metazoan species containing a nervous system, suggesting a beneficial purpose of sleep for even the simplest neural circuits. Local variations of sleep rhythms, i.e., specific to restrained portions of the mammalian brain, could account for neural circuit-specific sleep needs and sleep functions. In this review we use the term local sleep to refer to these local variations. During Non-Rapid-Eye-Movement (NREM) sleep, local sleep is now shown for major rhythms: slow-oscillation (SO, <∼1.5 Hz), delta (1.5–4 Hz) and sleep spindles (∼10–15 Hz). The SO is well-documented in rodents, cats, ferrets and humans, as a nesting oscillation composed of a silent and an active state, the latter carrying spindles and high frequency gamma rhythms (∼30–80 Hz) (Steriade et al., 1993; Achermann and Borbély, 1997; Rasch and Born, 2013; Halász et al., 2014; Neske, 2015; Crunelli et al., 2018). The cortical origin of the SO has been partly elucidated, in particular regarding the initiation, maintenance and termination of the active state (Sanchez-Vives and McCormick, 2000; Timofeev et al., 2000; Luczak et al., 2007; Sakata and Harris, 2009; Chauvette et al., 2010) (for review see, Neske, 2015). In contrast to the SO, delta waves are homeostatically regulated (Borbély and Tobler, 2011), and their generation mechanism are less clear—although the thalamus makes a strong contribution (Steriade et al., 1993; Halász et al., 2014; Crunelli et al., 2018; Fernandez et al., 2018). SO and delta, jointly described as a slow-wave activity (SWA, ∼0.5–4 Hz), show a decaying antero-posterior gradient in the human brain (Werth et al., 1997). SWA can also appear as single and seemingly independent events in local brain areas (Nir et al., 2011). Finally, sleep spindles can emerge in spatially confined regions, independently from the corresponding contralateral side (Nir et al., 2011), over regions probably corresponding to the size of functionally defined areas (Kim et al., 2015; Piantoni et al., 2017; Fernandez et al., 2018).

The description of local sleep in mammals is a fortunate development for endeavors attempting to dissect circuit functions of sleep. This is because developing techniques to identify and manipulate sleep locally might reveal directly the distinct assets of different sleep types for neural circuit properties, including in particular the role of one sleep rhythm over the others. An intriguing possibility is that local sleep is crucial for the maintenance and protection of the cellular and synaptic wiring of neural circuits and their plasticity. Prior studies have indeed proposed beneficial effects relevant for neural circuits such as: the regulation of synaptic strength and spine structure (Tononi and Cirelli, 2014; de Vivo et al., 2017), ion homeostasis of intracellular Ca^2+^ levels (Maret et al., 2007), cell-type-specific forms of excitability and plasticity (Kurotani et al., 2008; Astori et al., 2013), sensory receptive field regulation (Durkin et al., 2017), and undeniably more aspects still to be discovered. Local-sleep driven circuit reactivation also contributes to experience-dependent plasticity of local circuits (Huber et al., 2004; Bergmann et al., 2012; Johnson et al., 2012; Tamaki et al., 2013).

An influential hypothesis posits that wake-related area-specific increases in synaptic strength underlie local differences in SWA, suggesting that SWA takes on restorative functions in response to experience-dependent plasticity (Tononi and Cirelli, 2006). However, besides this hypothesis, so far there are no cellular or synaptic mechanisms known that underlie local differences in the spectral mix of sleep. Among the key players generating sleep patterns, there are the thalamus and the surrounding thalamic reticular nucleus (TRN) (Steriade and Llinas, 1988; Magnin et al., 2010; Halassa et al., 2014). The TRN is a peculiar shell-like inhibitory nucleus long known for its anatomical and functional heterogeneity. A large body of literature documents the role of the TRN in sleep rhythm generation, notably in sleep spindles, but also in delta and SO (Steriade and Llinas, 1988; Crunelli et al., 2018). However, despite its high heterogeneity and its crucial implication in sleep rhythms, the TRN contribution to local aspects of sleep has been considered only recently. This is even more surprising given that the TRN is strongly innervated by cortical inputs and is part of reciprocally connected and focalized thalamocortical loops, at least in sensory systems (Crabtree, 1999; Pinault, 2004). The cortical activity could drive the TRN, which would in return influence the cortex in a heterogeneous local manner.

Here, we explore how the TRN regulates sleep heterogeneity. Our review attempts to bring together the different anatomical, morphological and functional aspects of the TRN, disclosing a local circuit framework that shapes sleep spatial variability.

## Far From Being a Homogeneous Inhibitory Nucleus, the TRN Shows Anatomical, Morphological and Neurochemical Diversity

### Anatomical Diversity

**FIGURE 1:**
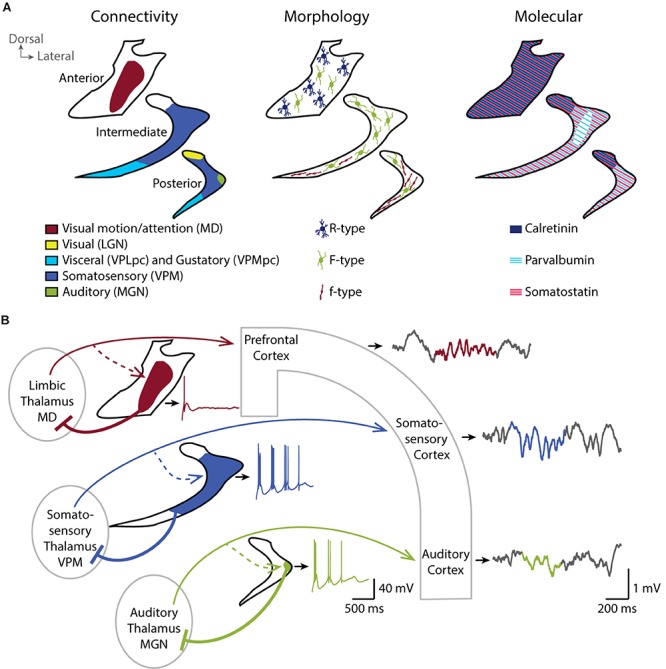
TheTRN is a heterogeneous nucleus and shapes local sleep through parallel thalamocortical loops. **(A)** Coronal schemes of the anterior, intermediate and posterior TRN showing the diversity of the connectivity (left), the repartition of TRN neurons with different morphological properties (middle), and the expression pattern of some of the major molecular markers (right). **(B)** Coronal scheme of three parallel thalamocortical loops showing the impact of distinct firing properties of TRN neurons in the different functional TRN sectors on the local activity of related cortices. Cortical inputs to the TRN are not depicted for clarity. *In vitro* whole-cell current-clamp traces of TRN neurons are shown next to the related TRN drawing (black outline). TRN neurons show rebound burst firing after a hyperpolarization step from –60 to –100 mV. Neurons in sensory sectors (green and blue) show more repetitive burst firing than the neuron in limbic sector (red). *In vivo* local field potentials simultaneously recorded in the different cortices during NREM sleep with detected spindle event (color highlighted) are shown on the right. Sensory cortices, in particular the somatosensory cortex in mice, are enriched in sleep spindles compared to prefrontal cortex. Whole-cell recording traces are adapted from Fernandez et al., 2018 (CC BY 4.0). MD, mediodorsal thalamic nucleus; VPLpc, Ventroposterolateral parvicellular thalamic nucleus; VPMpc, Ventroposteromedial parvicellular thalamic nucleus; LGN, Lateral geniculate thalamic nucleus; VPM, Ventroposteromedial thalamic nucleus; MGN, Medial geniculate thalamic nucleus.

The origin of cortical and subcortical afferents and the thalamic target of TRN neurons define anatomical subregions in the TRN. To describe these subregions, we focus here on the TRN’s thalamic targets. The postero-dorsal portion of the TRN is involved in visual (Crabtree and Killackey, 1989), auditory (Shosaku and Sumitomo, 1983) and somatosensory modalities (Crabtree, 1996). A gustatory sector (Hayama et al., 1994) and a visceral sector (Stehberg et al., 2001) overlap in the ventral portion of the TRN and extend from the posterior to the intermediate region along the anteroposterior axis. The anterior sector of the TRN is involved in motor and limbic structures (Gonzalo-Ruiz and Lieberman, 1995a, b; Zikopoulos and Barbas, 2007; Figure 1A, left). Primary sensory sectors of TRN (Crabtree, 1999) provide focalized and dense projections to first-order thalamic nuclei (Pinault and Deschênes, 1998), with the large majority of TRN cells targeting exactly one nucleus in a topographical order. The projections to higher-order thalamic sensory areas typically originate from the same sectors of TRN that target the first-order sensory areas, yet TRN cells projecting to higher-order nuclei have a final terminal arborization that extends over greater areas (Pinault et al., 1995). The TRN sector projecting to prefrontal-projecting thalamic nuclei occupies most of the rostral pole of the nucleus and is, at least in rodent, clearly segregated from the posterior sensory sectors. Projections into midline and intralaminar nuclei are made through small and less dense terminal arborizations compared to primary sensory projections (Pinault and Deschênes, 1998), with a less strict topographical organization (Cornwall et al., 1990; Kolmac and Mitrofanis, 1997).

### Morphological Diversity

Beyond this anatomical subdivision, early histological studies reveal several cytoarchitectonic and cellular differences in the TRN of ferret (Clemence and Mitrofanis, 1992), cat (Clemence and Mitrofanis, 1992; Lubke, 1993), rabbit (Lubke, 1993) and rat (Requena et al., 1991; Spreafico et al., 1991; Lubke, 1993). Spreafico undertakes a classification of three morphologically different types of neurons based on their soma shape, their dendritic arborization and their localization within the TRN (Figure 1A, middle). The R-type cells have a round soma and are preferentially located in anterior-limbic portions of TRN, whereas the small fusiform-shaped f-type cells are located in sensory sectors. Large fusiform F-type cells are found throughout the nucleus and have an axonal arborization within the TRN (Spreafico et al., 1988). Large F-type neurons receive chemical and electrical connections in similar proportion whereas small f-type seems to receive predominantly chemical synapses (Deleuze and Huguenard, 2006). The extent of the axonal arborization is also variable between TRN neurons. Cox et al. (1996) describe three patterns of projection into the ventrobasal thalamus of rat (cluster, intermediate and diffuse) and correlates the axonal arborization with the strength of inhibitory control over the thalamus (Cox et al., 1997). Diffuse axonal arborizations do not respect the boundaries of thalamic somatosensory vibrissae representation (called barreloids) and extend to a large region of the ventrobasal thalamus. On the contrary, neurons with clustered branching pattern form focal projections within barreloids, corresponding to the axonal arborizations described by Pinault and Deschênes (1998) and Desîlets-Roy et al. (2002). The intermediate axonal arborization covers an area fourfold greater than the clustered projection.

### Neurochemical Diversity

Additionally, the TRN neurons stain for a variety of neurochemical markers, most of which are not homogeneously distributed and not necessarily conserved across species. TRN cells stain homogeneously, and most prominently within the brain for the neurotransmitter gamma aminobutyric acid (GABA) (Penny et al., 1984), for Glutamic acid decarboxylase (GAD67) (Houser et al., 1980) and for vesicular transporter of GABA, such as VGAT2 (Halassa et al., 2011) from rodents to humans, consistent with the broad inhibitory synaptic transmission they convey to the thalamus. However, Ca^2+^-binding proteins such as calretinin and calbindin are not homogeneously expressed throughout the TRN. For example, calretinin and the KCl cotransporter KCC2 are preferentially expressed in the anterior and in the dorsal part of the rat TRN (Lizier et al., 1997; Bartho et al., 2004; Figure 1A, right). Calbindin-expressing neurons are instead found mostly in the posterior and intermediate regions of the rabbit TRN, corresponding to sensory sectors (Contreras-Rodriguez et al., 2003). The Ca^2+^-binding protein parvalbumin (PV) as well as the somatostatin (SOM)-expressing TRN neurons seem to form largely separate subpopulations that are intermingled throughout sectors, albeit to varying degrees (Figure 1A, right). For example, the inner tier of intermediate mouse TRN seems devoid of SOM-expressing neurons (Clemente-Perez et al., 2017). Other peptides, such as thyrotropin-releasing hormone (Segerson et al., 1987), vaso-active intestinal peptide (Burgunder et al., 1999), prolactin-releasing peptide (Roland et al., 1999) are also described in the rat TRN with disparate expression patterns throughout the nucleus (for review see, Pinault, 2004).

## Heterogeneous TRN-Thalamo-Cortical Functional Loops Shape Local Sleep in Cortex

### Sectorial Differences in TRN Cell Function

Our recent paper (Fernandez et al., 2018) is a first step towards demonstrating that TRN heterogeneity is essential to determine local sleep. We used optogenetically-assisted circuit mapping of TRN cell sectors in mice to define the functional properties of TRN cells based on their sectorial localization. In doing so, we identified both marked and graded differences in the endogenous discharge characteristics of TRN cells along sectors (Figure 1B). Cells in the somatosensory sector of the TRN showed the most vigorous endogenous repetitive bursting. The burst discharge of cells in the auditory sector of the TRN, while also pronounced, was weaker than in the somatosensory sector. In marked contrast, TRN cells innervated by the limbic mediodorsal thalamic nucleus failed to discharge in repetitive bursts. Burst differences are likely one of a number of several cellular and synaptic heterogeneities in TRN sectors that remain to be explored with respect to their impact on thalamic inhibition. In particular, whether these sectorial differences in discharge characteristics correspond to neurochemically distinct neuronal subtypes, such as PV- or SOM-expressing cells as described by Clemente-Perez remains to be determined (Clemente-Perez et al., 2017). Moreover, activity of TRN cells is differentially modulated depending on the sector of the TRN, visual or limbic, and the brain state, wakefulness or NREM sleep (Halassa et al., 2014). TRN-mediated inhibition of thalamic nuclei thus has unequal origins and might be recruited during different brain states and neuromodulatory conditions.

### Local Sleep Properties Correlate With Heterogeneity in TRN Burst Discharge

To determine whether sectorial differences in discharge patterns of TRN cells correspond to differences in local NREM sleep, we recorded multi-site local field potentials in the corresponding cortical areas (Figure 1B; Fernandez et al., 2018). These parallel recordings revealed marked differences in the local spectral composition of sleep. In particular, cortical somatosensory areas, that form loops with strongly bursting somatosensory TRN sector, produced fast and large spindles. This result is in line with previous findings in mice of prominent sigma power (10–15 Hz) in sensory-motor (Fernandez et al., 2017) and somatosensory regions (Kim et al., 2015). Intriguingly, human NREM sleep shows a clear sigma power peak and highest sleep spindle density in central derivations that include primary sensorimotor areas (Cox et al., 2017; Piantoni et al., 2017). Thus, some aspects of the spatial organization of NREM sleep spindles are similar in mice and humans. We next found that the power profile of local sleep in auditory and limbic prefrontal cortices does not contain such a strong sigma component, which correlates with the moderate repetitive bursting capacity of cells contained within the corresponding TRN sectors. Altogether, our findings offer evidence that heterogeneous cellular characteristics of TRN are linked to local sleep.

### Manipulating TRN Activity to Switch the Type of Local Sleep

To establish the causal relation between TRN bursting activity and local sleep properties in this study (Fernandez et al., 2018), we used either chemogenetic hyperpolarization of TRN cells, or genetic removal of the Ca_V_3.3 calcium channel, an essential element of TRN bursting (Astori et al., 2011; Pellegrini et al., 2016). Chemogenetic inhibition of TRN cells in the sensory sectors suppressed bursting and simultaneously hyperpolarized the cellular resting membrane potential, thus reducing excitability. Genetic removal of the Ca_V_3.3 channel suppressed bursting while maintaining regular firing. In both cases, local sleep in somatosensory cortex was largely depleted of sleep spindles, which indicates that repetitive bursting of TRN cells is the major determinant of sleep spindle density in these cortical areas. Instead, in prefrontal areas, in which spindles were less prominent, genetic removal of Ca_V_3.3 did not significantly affect the spectral composition of sleep nor of the properties of individual spindle events. Besides sleep spindles, the manipulation of TRN bursting also reduced power in the SO band, while upregulating delta power. Therefore, the TRN is important to regulate the spectral content of local sleep. These experiments open the possibility to modify local sleep by manipulating burst discharge of TRN sectors, for example through modulating the membrane potential of these cells.

Recent studies using optogenetic inhibition of TRN activity (Lewis et al., 2015; Herrera et al., 2016) also report alterations in the spectral characteristics of NREM sleep, yet without focusing on local variations in this specific vigilance state. Activating hypothalamic GABAergic input to the TRN switches the brain state from NREM sleep to wake (Herrera et al., 2016); while direct photoinhibition of the TRN in NREM sleep decreases the 1–4 Hz frequency band, but no distinction was made between the SO and delta range (Lewis et al., 2015). Our recent study finds in particular that local sleep in sensory cortex switches between spindle– and delta–enriched forms depending on the membrane potential of these cells. Multiple mechanisms regulate the membrane potential of TRN cells during natural sleep, in particular neuromodulation (Bal and McCormick, 1993; Ni et al., 2016) and activation of extrasynaptic NMDA receptors (Zhang et al., 2009). The exact conditions under which these are activated and whether there are sectorial differences remains to be elaborated.

## Implications of Thalamically–Driven Local Sleep for Local Neural Circuits

We explore here how experimental manipulations of TRN advance insights into the functions of local sleep, in particular when it comes to understanding its role in the maintenance and plasticity of neural circuits.

### Sleep Need of Local Neural Circuits

The amount of SWA during NREM sleep is homeostatically regulated according to the time spent awake, and primarily involves an increase in the delta component of SWA (Borbély and Tobler, 2011). SWA can also be upregulated locally in a cortical area according to its recent use. Somatosensory stimulation during wakefulness enhances SWA and spindles locally in the somatosensory areas in humans (Kattler et al., 1994), while SWA diminishes following a period of arm immobilization during wakefulness (Huber et al., 2006). Enhanced SWA indicates an enhanced need for sleep; therefore, somatosensory stimulation during the day augments the need of somatosensory thalamocortical loops to enter deep NREM sleep. Therefore, interference with local SWA increase after a period of sensory stimulation could help to explore local circuit alterations in a brain that otherwise sleeps normally. For example, intense sensory stimulation (e.g., whisker stimulation) during waking is known for enhancing SWA locally during NREM sleep in related sensory cortex (Vyazovskiy et al., 2000, 2004). Acute depolarization of TRN cells (Lewis et al., 2015; Fernandez et al., 2018) could be applied to see whether the use-dependent increase in the delta component of SWA can be prevented. Consequences on the functional and structural organization of the barrel cortex could then be traced to local sleep modification. This experimental approach could represent a step forward as it circumvents the non-specific effects of total sleep deprivation previously used to monitor circuit alterations in response to global homeostatic upregulation of NREM sleep. Moreover, it will unravel the distinct implication of the low-frequency (SO) and the high-frequency (delta) components of the SWA in local neural circuits.

### Developmental Regulation of Neural Circuits

A poorly explored aspect of local sleep is whether it is beneficial for the maturation and maintenance of neural connectivity. As considerably documented in the literature, local rhythmic activity occurs at early developmental stages in rodents (Tiriac and Blumberg, 2016) and in humans (Whitehead et al., 2017). Of particular interest is an immature form of spindle-related activity, the “neonatal spindle burst,” which results from self-generated muscle activity in the periphery, such as twitches of muscles or whiskers. The neonatal spindle burst is a predominant form of synchronized brain activity early in development (from late prenatal stages to the first postnatal weeks) and appears locally in cortical areas. These local rhythmic events are probably crucial for the establishment of sensorimotor maps (Del Rio-Bermudez and Blumberg, 2018). Although the relationship to mature sleep spindles is not clear, mechanistic similarities suggest that they could be forerunners (or at least immature signs) of the circuitries generating mature sleep spindles (Del Rio-Bermudez and Blumberg, 2018). Once the brain is adult, could local spindles continue to play a role in thalamocortical connectivity? This is supported by evidence that synchronized Ca^2+^ entry in thalamocortical cells—which also occurs during spindles—controls axonal outgrowth and synaptic refinement (Mire et al., 2012; Moreno-Juan et al., 2017). Furthermore, repetitive low-threshold bursting in TC cells induces waves of Ca^2+^ entry and increases in intracellular cAMP levels (Lüthi and McCormick, 1999) at frequencies that are known to serve as codes for growth cone responsiveness (Nicol et al., 2011). Barrel formation depends on the activity of adenylyl cyclase type I (AC I), a calcium-dependent version of the enzymes catalyzing synthesis of the intracellular messenger cAMP (Welker et al., 1996; Abdel-Majid et al., 1998; Lu et al., 2003). Disruption of the AC I-encoding gene results in the “barrelless” mouse model that lacks cortical barrels (Welker et al., 1996; Abdel-Majid et al., 1998). Loss of AC I in the sensory thalamus reproduces this developmental defect, disrupting cortical barrel patterns (Suzuki et al., 2015). To summarize, local sleep, by reactivating specific thalamocortical loops and triggering cAMP-dependent signaling cascades could maintain and reorganize connectivity of thalamocortical systems. Such possibilities can now be explored through manipulating local sleep via interfering with activity in TRN sectors.

## Conclusion and Perspectives

Local sleep, as spatial variations of sleep rhythms within the brain, is shaped by differences in thalamocortical connectivity rooted in the anatomy and functional activity of the TRN. In support of this, we summarize evidence that the TRN is segregated into different sectors with different cellular properties that tune the type of regional sleep patterns. We also argue that the TRN, due to the strong dependence of its discharge patterns on membrane potential, could be a versatile area to modify local sleep in response to use, experience and learning (Huber et al., 2004; Johnson et al., 2012; Tamaki et al., 2013). It remains to be seen whether compromised local neural circuits involving the TRN may produce aberrant local changes in the cortex. For example, a local wake state in motor areas is isolated in the sleeping brain in sleepwalking parasomniac patients (Terzaghi et al., 2009). Alternatively, in neuropsychotic diseases such as schizophrenia, strong deficits in sleep spindles possibly arise due to impaired TRN activity (Castelnovo et al., 2017; Thankachan et al., 2019), pointing to the hypothesis that sleep rhythm abnormalities in these diseases could be related to aberrant activity in the local sectors of the TRN. Dysregulation and abnormal compartmentalization of sleep could impact other diseases for which the link has not yet been established, compromising brain connectivity and learning processes. Further understanding of the mechanisms regulating the TRN will be essential in delineating the importance of the spatial heterogeneity of NREM sleep for local neural circuits in restorative and cognitive functions.

## Author Contributions

All authors wrote, read, and corrected the manuscript. GV and LF elaborated the figure.

## Conflict of Interest Statement

The authors declare that the research was conducted in the absence of any commercial or financial relationships that could be construed as a potential conflict of interest.

## References

[B1] Abdel-Majid R. M. Leong W. L. Schalkwyk L. C. Smallman D. S. Wong S. T. Storm D. R. (1998). Loss of adenylyl cyclase I activity disrupts patterning of mouse somatosensory cortex. *Nat. Genet.* 19 289–291. 10.1038/980 9662407

[B2] Achermann P. Borbély A. A. (1997). Low-frequency (< 1 Hz) oscillations in the human sleep electroencephalogram. *Neuroscience* 81 213–222. 10.1016/s0306-4522(97)00186-3 9300413

[B3] Astori S. Wimmer R. D. Luthi A. (2013). Manipulating sleep spindles—expanding views on sleep, memory, and disease. *Trends Neurosci.* 36 738–748. 10.1016/j.tins.2013.10.001 24210901

[B4] Astori S. Wimmer R. D. Prosser H. M. Corti C. Corsi M. Liaudet N. (2011). The CaV3.3 calcium channel is the major sleep spindle pacemaker in thalamus. *Proc. Natl. Acad. Sci. U.S.A.* 108 13823–13828. 10.1073/pnas.1105115108 21808016 PMC3158184

[B5] Bal T. McCormick D. A. (1993). Mechanisms of oscillatory activity in guinea-pig nucleus reticularis thalami in vitro: a mammalian pacemaker. *J. Physiol.* 468 669–691. 10.1113/jphysiol.1993.sp019794 8254530 PMC1143849

[B6] Bartho P. Payne J. A. Freund T. F. Acsady L. (2004). Differential distribution of the KCl cotransporter KCC2 in thalamic relay and reticular nuclei. *Eur. J. Neurosci.* 20 965–975. 10.1111/j.1460-9568.2004.03562.x 15305865 PMC2630852

[B7] Bergmann T. O. Mölle M. Diedrichs J. Born J. Siebner H. R. (2012). Sleep spindle-related reactivation of category-specific cortical regions after learning face-scene associations. *Neuroimage* 59 2733–2742. 10.1016/j.neuroimage.2011.10.036 22037418

[B8] Borbély A. A. Tobler I. (2011). Manifestations and functional implications of sleep homeostasis. *Handb. Clin. Neurol.* 98 205–213. 10.1016/b978-0-444-52006-7.00013-7 21056188

[B9] Burgunder J. M. Heyberger B. Lauterburg T. (1999). Thalamic reticular nucleus parcellation delineated by VIP and TRH gene expression in the rat. *J. Chem. Neuroanat.* 17 147–152. 10.1016/s0891-0618(99)00033-2 10609863

[B10] Castelnovo A. Graziano B. Ferrarelli F. D’Agostino A. (2017). Sleep spindles and slow waves in schizophrenia and related disorders: main findings, challenges and future perspectives. *Eur. J. Neurosci.* 48 2738–2758. 10.1111/ejn.13815 29280209

[B11] Chauvette S. Volgushev M. Timofeev I. (2010). Origin of active states in local neocortical networks during slow sleep oscillation. *Cereb. Cortex* 20 2660–2674. 10.1093/cercor/bhq009 20200108 PMC2951844

[B12] Clemence A. E. Mitrofanis J. (1992). Cytoarchitectonic heterogeneities in the thalamic reticular nucleus of cats and ferrets. *J. Comp. Neurol.* 322 167–180. 10.1002/cne.903220203 1381730

[B13] Clemente-Perez A. Makinson S. R. Higashikubo B. Brovarney S. Cho F. S. Urry A. (2017). Distinct thalamic reticular cell types differentially modulate normal and pathological cortical rhythms. *Cell Rep.* 19 2130–2142. 10.1016/j.celrep.2017.05.044 28591583 PMC5557038

[B14] Contreras-Rodriguez J. Gonzalez-Soriano J. Martinez-Sainz P. Marin-Garcia P. Rodriguez-Veiga E. (2003). Neurochemical heterogeneity of the thalamic reticular and perireticular nuclei in developing rabbits: patterns of calbindin expression. *Brain Res. Dev. Brain Res.* 144 211–221. 10.1016/s0165-3806(03)00194-9 12935918

[B15] Cornwall J. Cooper J. D. Phillipson O. T. (1990). Projections to the rostral reticular thalamic nucleus in the rat. *Exp. Brain Res.* 80 157–171. 2358025 10.1007/BF00228857

[B16] Cox C. L. Huguenard J. R. Prince D. A. (1996). Heterogeneous axonal arborizations of rat thalamic reticular neurons in the ventrobasal nucleus. *J. Comp. Neurol.* 366 416–430. 10.1002/(sici)1096-9861(19960311)366:3<416::aid-cne4>3.0.co;2-7 8907356

[B17] Cox C. L. Huguenard J. R. Prince D. A. (1997). Nucleus reticularis neurons mediate diverse inhibitory effects in thalamus. *Proc. Natl. Acad. Sci. U.S.A.* 94 8854–8859. 10.1073/pnas.94.16.8854 9238067 PMC23165

[B18] Cox R. Schapiro A. C. Manoach D. S. Stickgold R. (2017). Individual differences in frequency and topography of slow and fast sleep spindles. *Front. Hum Neurosci.* 11:433. 10.3389/fnhum.2017.00433 28928647 PMC5591792

[B19] Crabtree J. W. (1996). Organization in the somatosensory sector of the cat’s thalamic reticular nucleus. *J. Comp. Neurol.* 366 207–222. 10.1002/(sici)1096-9861(19960304)366:2<207::aid-cne2>3.3.co;2-m 8698882

[B20] Crabtree J. W. (1999). Intrathalamic sensory connections mediated by the thalamic reticular nucleus. *Cell Mol. Life. Sci.* 56 683–700. 10.1007/s000180050462 11212315 PMC11146980

[B21] Crabtree J. W. Killackey H. P. (1989). The topographic organization and axis of projection within the visual sector of the rabbit’s thalamic reticular nucleus. *Eur. J. Neurosci.* 1 94–109. 10.1111/j.1460-9568.1989.tb00777.x 12106177

[B22] Crunelli V. Lörincz M. L. Connelly W. M. David F. Hughes S. W. Lambert R. C. (2018). Dual function of thalamic low-vigilance state oscillations: rhythm-regulation and plasticity. *Nat. Rev. Neurosci.* 19 107–118. 10.1038/nrn.2017.151 29321683 PMC6364803

[B23] de Vivo L. Bellesi M. Marshall W. Bushong E. A. Ellisman M. H. Tononi G. (2017). Ultrastructural evidence for synaptic scaling across the wake/sleep cycle. *Science* 355 507–510. 10.1126/science.aah5982 28154076 PMC5313037

[B24] Del Rio-Bermudez C. Blumberg M. S. (2018). Active sleep promotes functional connectivity in developing sensorimotor networks. *Bioessays* 40:e1700234. 10.1002/bies.201700234 29508913 PMC6247910

[B25] Deleuze C. Huguenard J. R. (2006). Distinct electrical and chemical connectivity maps in the thalamic reticular nucleus: potential roles in synchronization and sensation. *J. Neurosci.* 26 8633–8645. 10.1523/jneurosci.2333-06.2006 16914689 PMC6674339

[B26] Desîlets-Roy B. Varga C. Lavallée P. Deschênes M. (2002). Substrate for cross-talk inhibition between thalamic barreloids. *J. Neurosci.* 22:RC218. 11978859 10.1523/JNEUROSCI.22-09-j0002.2002 PMC6758391

[B27] Durkin J. Suresh A. K. Colbath J. Broussard C. Wu J. Zochowski M. (2017). Cortically coordinated NREM thalamocortical oscillations play an essential, instructive role in visual system plasticity. *Proc. Natl. Acad. Sci. U.S.A.* 114 10485–10490. 10.1073/pnas.1710613114 28893999 PMC5625927

[B28] Fernandez L. M. Vantomme G. Osorio-Forero A. Cardis R. Béard E. Lüthi A. (2018). Thalamic reticular control of local sleep in mouse sensory cortex. *Elife* 7:e39111. 10.7554/eLife.39111 30583750 PMC6342525

[B29] Fernandez L. M. J. Comte J. C. Le Merre P. Lin J. S. Salin P. A. Crochet S. (2017). Highly dynamic spatiotemporal organization of low-frequency activities during behavioral states in the mouse cerebral cortex. *Cereb. Cortex* 27 5444–5462. 10.1093/cercor/bhw311 27742711

[B30] Gonzalo-Ruiz A. Lieberman A. R. (1995a). GABAergic projections from the thalamic reticular nucleus to the anteroventral and anterodorsal thalamic nuclei of the rat. *J. Chem. Neuroanat.* 9 165–174. 10.1016/0891-0618(95)00078-x 8588832

[B31] Gonzalo-Ruiz A. Lieberman A. R. (1995b). Topographic organization of projections from the thalamic reticular nucleus to the anterior thalamic nuclei in the rat. *Brain Res. Bull.* 37 17–35. 10.1016/0361-9230(94)00252-5 7606476

[B32] Halassa M. M. Chen Z. Wimmer R. D. Brunetti P. M. Zhao S. Zikopoulos B. (2014). State-dependent architecture of thalamic reticular subnetworks. *Cell* 158 808–821. 10.1016/j.cell.2014.06.025 25126786 PMC4205482

[B33] Halassa M. M. Siegle J. H. Ritt J. T. Ting J. T. Feng G. Moore C. I. (2011). Selective optical drive of thalamic reticular nucleus generates thalamic bursts and cortical spindles. *Nat. Neurosci.* 14 1118–1120. 10.1038/nn.2880 21785436 PMC4169194

[B34] Halász P. Bódizs R. Parrino L. Terzano M. (2014). Two features of sleep slow waves: homeostatic and reactive aspects–from long term to instant sleep homeostasis. *Sleep Med.* 15 1184–1195. 10.1016/j.sleep.2014.06.006 25192672

[B35] Hayama T. Hashimoto K. Ogawa H. (1994). Anatomical location of a taste-related region in the thalamic reticular nucleus in rats. *Neurosci. Res.* 18 291–299. 10.1016/0168-0102(94)90165-1 7514778

[B36] Herrera C. G. Cadavieco M. C. Jego S. Ponomarenko A. Korotkova T. Adamantidis A. (2016). Hypothalamic feedforward inhibition of thalamocortical network controls arousal and consciousness. *Nat. Neurosci.* 19 290–298. 10.1038/nn.4209 26691833 PMC5818272

[B37] Houser C. R. Vaughn J. E. Barber R. P. Roberts E. (1980). GABA neurons are the major cell type of the nucleus reticularis thalami. *Brain Res.* 200 341–354. 10.1016/0006-8993(80)90925-7 7417821

[B38] Huber R. Ghilardi M. F. Massimini M. Ferrarelli F. Riedner B. A. Peterson M. J. (2006). Arm immobilization causes cortical plastic changes and locally decreases sleep slow wave activity. *Nat. Neurosci.* 9 1169–1176. 10.1038/nn1758 16936722

[B39] Huber R. Ghilardi M. F. Massimini M. Tononi G. (2004). Local sleep and learning. *Nature* 430 78–81. 10.1038/nature02663 15184907

[B40] Johnson L. A. Blakely T. Hermes D. Hakimian S. Ramsey N. F. Ojemann J. G. (2012). Sleep spindles are locally modulated by training on a brain-computer interface. *Proc. Natl. Acad. Sci. U.S.A.* 109 18583–18588. 10.1073/pnas.1207532109 23091013 PMC3494921

[B41] Kattler H. Dijk D. J. Borbély A. A. (1994). Effect of unilateral somatosensory stimulation prior to sleep on the sleep EEG in humans. *J. Sleep Res.* 3 159–164. 10.1111/j.1365-2869.1994.tb00123.x 10607121

[B42] Kim D. Hwang E. Lee M. Sung H. Choi J. H. (2015). Characterization of topographically specific sleep spindles in mice. *Sleep* 38 85–96. 10.5665/sleep.4330 25325451 PMC4262960

[B43] Kolmac C. I. Mitrofanis J. (1997). Organisation of the reticular thalamic projection to the intralaminar and midline nuclei in rats. *J. Comp. Neurol.* 377 165–178. 10.1002/(sici)1096-9861(19970113)377:2<165::aid-cne2>3.0.co;2-1 8986879

[B44] Kurotani T. Yamada K. Yoshimura Y. Crair M. C. Komatsu Y. (2008). State-dependent bidirectional modification of somatic inhibition in neocortical pyramidal cells. *Neuron* 57 905–916. 10.1016/j.neuron.2008.01.030 18367091 PMC2880402

[B45] Lewis L. D. Voigts J. Flores F. J. Schmitt L. I. Wilson M. A. Halassa M. M. (2015). Thalamic reticular nucleus induces fast and local modulation of arousal state. *Elife* 4:e08760. 10.7554/eLife.08760 26460547 PMC4686423

[B46] Lizier C. Spreafico R. Battaglia G. (1997). Calretinin in the thalamic reticular nucleus of the rat: distribution and relationship with ipsilateral and contralateral efferents. *J. Comp. Neurol.* 377 217–233. 10.1002/(sici)1096-9861(19970113)377:2<217::aid-cne5>3.0.co;2-6 8986882

[B47] Lu H. C. She W. C. Plas D. T. Neumann P. E. Janz R. Crair M. C. (2003). Adenylyl cyclase I regulates AMPA receptor trafficking during mouse cortical ‘barrel’ map development. *Nat. Neurosci.* 6 939–947. 10.1038/nn1106 12897788

[B48] Lubke J. (1993). Morphology of neurons in the thalamic reticular nucleus (TRN) of mammals as revealed by intracellular injections into fixed brain slices. *J. Comp. Neurol.* 329 458–471. 10.1002/cne.903290404 8454736

[B49] Luczak A. Bartho P. Marguet S. L. Buzsaki G. Harris K. D. (2007). Sequential structure of neocortical spontaneous activity in vivo. *Proc. Natl. Acad. Sci. U.S.A.* 104 347–352. 10.1073/pnas.0605643104 17185420 PMC1765463

[B50] Lüthi A. McCormick D. A. (1999). Modulation of a pacemaker current through Ca2+-induced stimulation of cAMP production. *Nat. Neurosci.* 2 634–641. 10.1038/10189 10404196

[B51] Magnin M. Rey M. Bastuji H. Guillemant P. Mauguiere F. Garcia-Larrea L. (2010). Thalamic deactivation at sleep onset precedes that of the cerebral cortex in humans. *Proc. Natl. Acad. Sci. U.S.A.* 107 3829–3833. 10.1073/pnas.0909710107 20142493 PMC2840430

[B52] Maret S. Dorsaz S. Gurcel L. Pradervand S. Petit B. Pfister C. (2007). Homer1a is a core brain molecular correlate of sleep loss. *Proc. Natl. Acad. Sci. U.S.A.* 104 20090–20095. 10.1073/pnas.0710131104 18077435 PMC2148427

[B53] Mire E. Mezzera C. Leyva-Díaz E. Paternain A. V. Squarzoni P. Bluy L. (2012). Spontaneous activity regulates robo1 transcription to mediate a switch in thalamocortical axon growth. *Nat. Neurosci.* 15 1134–1143. 10.1038/nn.3160 22772332

[B54] Moreno-Juan V. Filipchuk A. Antón-Bolaños N. Mezzera C. Gezelius H. Andrés B. (2017). Prenatal thalamic waves regulate cortical area size prior to sensory processing. *Nat. Commun.* 8:14172. 10.1038/ncomms14172 28155854 PMC5296753

[B55] Nath R. D. Bedbrook C. N. Abrams M. J. Basinger T. Bois J. S. Prober D. A. (2017). The jellyfish Cassiopea exhibits a sleep-like state. *Curr. Biol.* 27:e2983. 10.1016/j.cub.2017.08.014 28943083 PMC5653286

[B56] Neske G. T. (2015). The slow oscillation in cortical and thalamic networks: mechanisms and functions. *Front. Neural Circ.* 9:88. 10.3389/fncir.2015.00088 26834569 PMC4712264

[B57] Ni K. M. Hou X. J. Yang C. H. Dong P. Li Y. Zhang Y. (2016). Selectively driving cholinergic fibers optically in the thalamic reticular nucleus promotes sleep. *Elife* 5:e10382. 10.7554/eLife.10382 26880556 PMC4764559

[B58] Nicol X. Hong K. P. Spitzer N. C. (2011). Spatial and temporal second messenger codes for growth cone turning. *Proc. Natl. Acad. Sci. U.S.A.* 108 13776–13781. 10.1073/pnas.1100247108 21795610 PMC3158175

[B59] Nir Y. Staba R. J. Andrillon T. Vyazovskiy V. V. Cirelli C. Fried I. (2011). Regional slow waves and spindles in human sleep. *Neuron* 70 153–169. 10.1016/j.neuron.2011.02.043 21482364 PMC3108825

[B60] Pellegrini C. Lecci S. Lüthi A. Astori S. (2016). Suppression of sleep spindle rhythmogenesis in mice with deletion of CaV3.2 and CaV3.3 T-type Ca2+ channels. *Sleep* 39 875–885. 10.5665/sleep.5646 26612388 PMC4791621

[B61] Penny G. R. Conley M. Schmechel D. E. Diamond I. T. (1984). The distribution of glutamic acid decarboxylase immunoreactivity in the diencephalon of the opossum and rabbit. *J. Comp. Neurol.* 228 38–56. 10.1002/cne.902280106 6090511

[B62] Piantoni G. Halgren E. Cash S. S. (2017). Spatiotemporal characteristics of sleep spindles depend on cortical location. *Neuroimage* 146 236–245. 10.1016/j.neuroimage.2016.11.010 27840241 PMC5321858

[B63] Pinault D. (2004). The thalamic reticular nucleus: structure, function and concept. *Brain Res. Brain Res. Rev.* 46 1–31. 10.1016/j.brainresrev.2004.04.008 15297152

[B64] Pinault D. Bourassa J. Deschênes M. (1995). The axonal arborization of single thalamic reticular neurons in the somatosensory thalamus of the rat. *Eur. J. Neurosci.* 7 31–40. 10.1111/j.1460-9568.1995.tb01017.x 7711934

[B65] Pinault D. Deschênes M. (1998). Projection and innervation patterns of individual thalamic reticular axons in the thalamus of the adult rat: a three-dimensional, graphic, and morphometric analysis. *J. Comp. Neurol.* 391 180–203. 10.1002/(sici)1096-9861(19980209)391:2<180::aid-cne3>3.0.co;2-z 9518268

[B66] Rasch B. Born J. (2013). About sleep’s role in memory. *Physiol. Rev.* 93 681–766. 10.1152/physrev.00032.2012 23589831 PMC3768102

[B67] Requena V. Diaz F. Villena A. Vidal L. Perez de Vargas I. (1991). Histoenzymatic study of the neurons in the visual sector of the thalamic reticular nucleus in the adult rat. *J. Hirnforsch.* 32 459–467. 1802930

[B68] Roland B. L. Sutton S. W. Wilson S. J. Luo L. Pyati J. Huvar R. (1999). Anatomical distribution of prolactin-releasing peptide and its receptor suggests additional functions in the central nervous system and periphery. *Endocrinology* 140 5736–5745. 10.1210/en.140.12.5736 10579339

[B69] Sakata S. Harris K. D. (2009). Laminar structure of spontaneous and sensory-evoked population activity in auditory cortex. *Neuron* 64 404–418. 10.1016/j.neuron.2009.09.020 19914188 PMC2778614

[B70] Sanchez-Vives M. V. McCormick D. A. (2000). Cellular and network mechanisms of rhythmic recurrent activity in neocortex. *Nat. Neurosci.* 3 1027–1034. 10.1038/79848 11017176

[B71] Segerson T. P. Hoefler H. Childers H. Wolfe H. J. Wu P. Jackson I. M. (1987). Localization of thyrotropin-releasing hormone prohormone messenger ribonucleic acid in rat brain in situ hybridization. *Endocrinology* 121 98–107. 10.1210/endo-121-1-98 3109882

[B72] Shosaku A. Sumitomo I. (1983). Auditory neurons in the rat thalamic reticular nucleus. *Exp. Brain Res.* 49 432–442. 6641840 10.1007/BF00238784

[B73] Spreafico R. Battaglia G. Frassoni C. (1991). The reticular thalamic nucleus (RTN) of the rat: cytoarchitectural, Golgi, immunocytochemical, and horseradish peroxidase study. *J. Comp. Neurol.* 304 478–490. 10.1002/cne.903040311 1708789

[B74] Spreafico R. de Curtis M. Frassoni C. Avanzini G. (1988). Electrophysiological characteristics of morphologically identified reticular thalamic neurons from rat slices. *Neuroscience* 27 629–638. 10.1016/0306-4522(88)90294-1 3217007

[B75] Stehberg J. Acuna-Goycolea C. Ceric F. Torrealba F. (2001). The visceral sector of the thalamic reticular nucleus in the rat. *Neuroscience* 106 745–755. 10.1016/s0306-4522(01)00316-5 11682160

[B76] Steriade M. Llinas R. R. (1988). The functional states of the thalamus and the associated neuronal interplay. *Physiol. Rev.* 68 649–742. 10.1152/physrev.1988.68.3.649 2839857

[B77] Steriade M. McCormick D. A. Sejnowski T. J. (1993). Thalamocortical oscillations in the sleeping and aroused brain. *Science* 262 679–685. 10.1126/science.8235588 8235588

[B78] Suzuki A. Lee L. J. Hayashi Y. Muglia L. Itohara S. Erzurumlu R. S. (2015). Thalamic adenylyl cyclase 1 is required for barrel formation in the somatosensory cortex. *Neuroscience* 290 518–529. 10.1016/j.neuroscience.2015.01.043 25644422 PMC5994750

[B79] Tamaki M. Huang T. R. Yotsumoto Y. Hämäläinen M. Lin F. H. Náñez J. E. Sr. (2013). Enhanced spontaneous oscillations in the supplementary motor area are associated with sleep-dependent offline learning of finger-tapping motor-sequence task. *J. Neurosci.* 33 13894–13902. 10.1523/JNEUROSCI.1198-13.2013 23966709 PMC3755724

[B80] Terzaghi M. Sartori I. Tassi L. Didato G. Rustioni V. LoRusso G. (2009). Evidence of dissociated arousal states during NREM parasomnia from an intracerebral neurophysiological study. *Sleep* 32 409–412. 10.1093/sleep/32.3.409 19294961 PMC2647795

[B81] Thankachan S. Katsuki F. McKenna J. T. Yang C. Shukla C. Deisseroth K. (2019). Thalamic reticular nucleus parvalbumin neurons regulate sleep spindles and electrophysiological aspects of schizophrenia in mice. *Sci. Rep.* 9:3607. 10.1038/s41598-019-40398-9 30837664 PMC6401113

[B82] Timofeev I. Grenier F. Bazhenov M. Sejnowski T. J. Steriade M. (2000). Origin of slow cortical oscillations in deafferented cortical slabs. *Cereb. Cortex* 10 1185–1199. 10.1093/cercor/10.12.1185 11073868

[B83] Tiriac A. Blumberg M. S. (2016). The case of the disappearing spindle burst. *Neural Plast.* 2016:8037321. 10.1155/2016/8037321 27119028 PMC4826930

[B84] Tononi G. Cirelli C. (2006). Sleep function and synaptic homeostasis. *Sleep Med. Rev.* 10 49–62. 10.1016/j.smrv.2005.05.002 16376591

[B85] Tononi G. Cirelli C. (2014). Sleep and the price of plasticity: from synaptic and cellular homeostasis to memory consolidation and integration. *Neuron* 81 12–34. 10.1016/j.neuron.2013.12.025 24411729 PMC3921176

[B86] Vyazovskiy V. Borbély A. A. Tobler I. (2000). Unilateral vibrissae stimulation during waking induces interhemispheric EEG asymmetry during subsequent sleep in the rat. *J. Sleep Res.* 9 367–371. 10.1046/j.1365-2869.2000.00230.x 11123523

[B87] Vyazovskiy V. V. Welker E. Fritschy J. M. Tobler I. (2004). Regional pattern of metabolic activation is reflected in the sleep EEG after sleep deprivation combined with unilateral whisker stimulation in mice. *Eur. J. Neurosci.* 20 1363–1370. 10.1111/j.1460-9568.2004.03583.x 15341608

[B88] Welker E. Armstrong-James M. Bronchti G. Ourednik W. Gheorghita-Baechler F. Dubois R. (1996). Altered sensory processing in the somatosensory cortex of the mouse mutant barrelless. *Science* 271 1864–1867. 10.1126/science.271.5257.1864 8596955

[B89] Werth E. Achermann P. Borbély A. A. (1997). Fronto-occipital EEG power gradients in human sleep. *J. Sleep Res.* 6 102–112. 10.1046/j.1365-2869.1997.d01-36.x 9377529

[B90] Whitehead K. Pressler R. Fabrizi L. (2017). Characteristics and clinical significance of delta brushes in the EEG of premature infants. *Clin. Neurophysiol. Pract.* 2 12–18. 10.1016/j.cnp.2016.11.002 30214965 PMC6123866

[B91] Zhang Y. Llinas R. R. Lisman J. E. (2009). Inhibition of NMDARs in the nucleus reticularis of the thalamus produces delta frequency bursting. *Front. Neural Circ.* 3:20. 10.3389/neuro.04.020.2009 20057928 PMC2802545

[B92] Zikopoulos B. Barbas H. (2007). Circuits formultisensory integration and attentional modulation through the prefrontal cortex and the thalamic reticular nucleus in primates. *Rev. Neurosci.* 18 417–438. 18330211 10.1515/revneuro.2007.18.6.417 PMC2855189

